# Reduced Graphene Oxide on Nickel Foam for Supercapacitor Electrodes

**DOI:** 10.3390/ma10111295

**Published:** 2017-11-11

**Authors:** Uma Ramabadran, Gillian Ryan, Xuan Zhou, Susan Farhat, Felicia Manciu, Yigang Tong, Ryan Ayler, Graham Garner

**Affiliations:** 1 Department of Physics, Kettering University, Flint, MI 48504, USA; gryan@kettering.edu; 2 Department of Electrical and Computer Engineering, Kettering University, Flint, MI 48504, USA; xzhou@kettering.edu (X.Z.); tong9285@kettering.edu (Y.T.); 3 Department of Chemical Engineering, Kettering University, Flint, MI 48504, USA; sfarhat@kettering.edu (S.F.); garn9198@kettering.edu (G.G.); 4 Department of Physics, University of Texas at El Paso, El Paso, TX 79968, USA; fsmanciu@utep.edu; 5 Department of Mechanical Engineering, Kettering University, Flint, MI 48504, USA; rayler@gmail.com

**Keywords:** RGO, nickel foam, atmospheric plasma, furnace processing, graphene, electrodes

## Abstract

The focus of this paper is the investigation of reduced graphene oxide (GO)/nickel foam (RGON) samples for use as supercapacitor electrodes. Nickel foam samples were soaked in a GO suspension and dried before being subjected to two different methods to remove oxygen. Atmospheric pressure annealed (APA) samples were treated with a varying number (10–18) of nitrogen plasma jet scans, where sample temperatures did not exceed 280 °C. Furnace annealed (FA) samples were processed in an atmosphere of hydrogen and argon, at temperatures ranging from 600 °C to 900 °C. Environmental Scanning Electron Microscope (ESEM) data indicated that the carbon to oxygen (C:O) ratio for APA samples was minimized at an intermediate number of plasma scans. Fourier Transform Infrared Spectroscopic (FTIR) and Raman spectroscopic data supported this finding. ESEM analysis from FA samples showed that with increasing temperatures of annealing, GO is transformed to reduced graphene oxide (RGO), with C:O ratios exceeding 35:1. X-ray Photoelectron Spectroscopy (XPS) and X-ray diffraction (XRD) data indicated the formation of RGO with an increasing annealing temperature until 800 °C, when oxygen reincorporation in the surface atomic layers becomes an issue. Supercapacitors, constructed using the FA samples, demonstrated performances that correlated with surface atomic layer optimization of the C:O ratio.

## 1. Introduction

Supercapacitors have rapid charging and discharging abilities at high power densities and can be cycled thousands of times without significant degradation. Typical energy densities of supercapacitors are a million times higher than that of conventional capacitors. They can also store and deliver energy at considerably higher rates compared to rechargeable batteries. As a result, they have generated great interest in a growing range of applications, such as forklifts, load cranes, electric vehicles and power back-up in industries [1,2,3,4]. To construct these supercapacitors, highly conducting, large surface area electrodes are required.

Porous carbon, multi-walled carbon nanotubes (MWCNT), single-walled carbon nanotubes (SWCNT), and hybrid materials have been used as supercapacitor electrodes [5,6,7,8,9]. Although these materials offer high surface areas, adhesion issues and high contact resistances between the electrodes and current collectors remains an issue, and their low conductivity limits their energy application [10]. Graphene, which has a one atom thick, two-dimensional (2D) structure, has displayed an excellent electrochemical energy storage ability, primarily due to its conducting properties and chemical stability. The ballistic electron mobility exhibited by graphene at room temperature, along with its high surface area and tensile strength suggests that it may be an attractive electrode material for supercapacitors. Methods such as exfoliation [11] and chemical vapor deposition (CVD) [12] have been employed to make graphene films a few atomic layers thick. The challenge for implementation of graphene-based electrodes has been to fabricate films of reasonable size inexpensively on suitable substrates. Removing oxygen from graphene oxide (GO) results in the formation of reduced graphene oxide (RGO). This material, prepared by chemical reduction, has reported conductivities of thousands of Seimens/meter, and can be obtained at a reasonable cost [13,14,15].

RGO has been produced by other techniques, such as laser scribing, electron beam irradiation and thermal processing, on substrates, such as aluminum, copper and single crystal nickel [16,17,18,19]. GO on nickel foam (RGON) was reported to have better properties than commonly used electrodes made of composites of gold or platinum with polymer binders [20]. Additionally, chemically-reduced graphene oxide hydrogels as well as novel nickel oxide/graphene composites using CVD have also been fabricated for electrodes [21,22].

In this paper we outline results obtained from the investigation of reduced graphene oxide/nickel foam (RGON) samples for use as supercapacitor electrodes. The sample electrodes were fabricated by soaking nickel foam in a GO suspension, air-drying them and subjecting them to two different processes aimed at removing oxygen. One set of samples were subject to a scanning jet of compressed air containing ionized nitrogen cold plasma with a high kinetic energy. This low temperature atmospheric plasma annealing (APA) process offers a relatively inexpensive method for electrode fabrication, eliminating the need for a binder. The result is the removal of oxygen from the GO by nitrogen from the plasma, thereby forming RGO. This technique enables rapid reduction of GO without the use of toxic chemicals. The results from these experiments were compared to those obtained from samples that were subjected to a proven furnace processing technique, which had a mixture of argon and hydrogen flowing through it [23]. We refer to these samples as having been furnace annealed (FA). The RGON electrode materials, fabricated by the APA and FA processes, were analyzed using environmental scanning electron microscopy (ESEM), X-ray photoemission spectroscopy (XPS), Fourier transform infrared spectroscopy (FTIR) and Raman spectroscopy. The results established that RGO can be obtained by APA. However, the APA samples were mechanically fragile and therefore XRD and capacitance characterization was not possible. Further work is planned to address this issue. XRD data was obtained from the samples fabricated by FA, which supported the conclusion that RGO is formed. These RGON electrodes were used to construct electrical double layer capacitors (EDLC). Preliminary data obtained from the capacitors suggests that oxygen content on the surface layers correlates with capacitor performance.

## 2. Results and Discussion

### 2.1. Environmental Scanning Electron Microscopy (ESEM) Results

SEM images were obtained using a Quanta 200s system (Thermo Fisher Scientific, Waltham, MA, USA) from an unprocessed RGON sample, as well as the samples that were subject to APA. The chemical composition over randomly selected areas was also determined at different locations on the sample using the Energy Dispersive X-ray (EDX) analysis feature. The ratio of carbon to oxygen was calculated by averaging values over 3 or more such areas, which were over hundreds of square micrometers in size.

**Figure 1:**
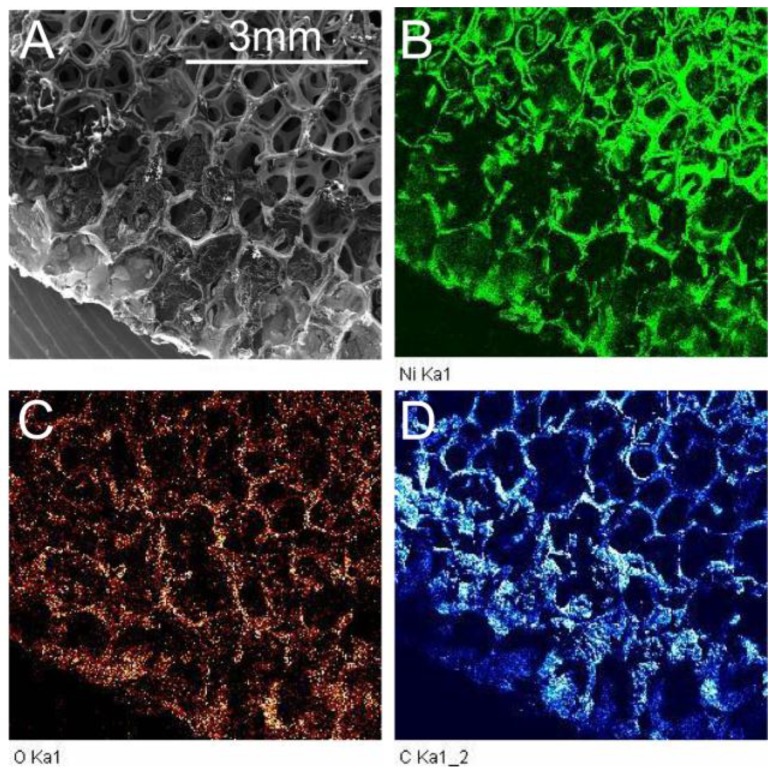
SEM image (**A**), chemical map of nickel (**B**), oxygen (**C**) and carbon (**D**) from a sample with 12 Atmospheric pressure annealed (APA) scans.

**Figure 2:**
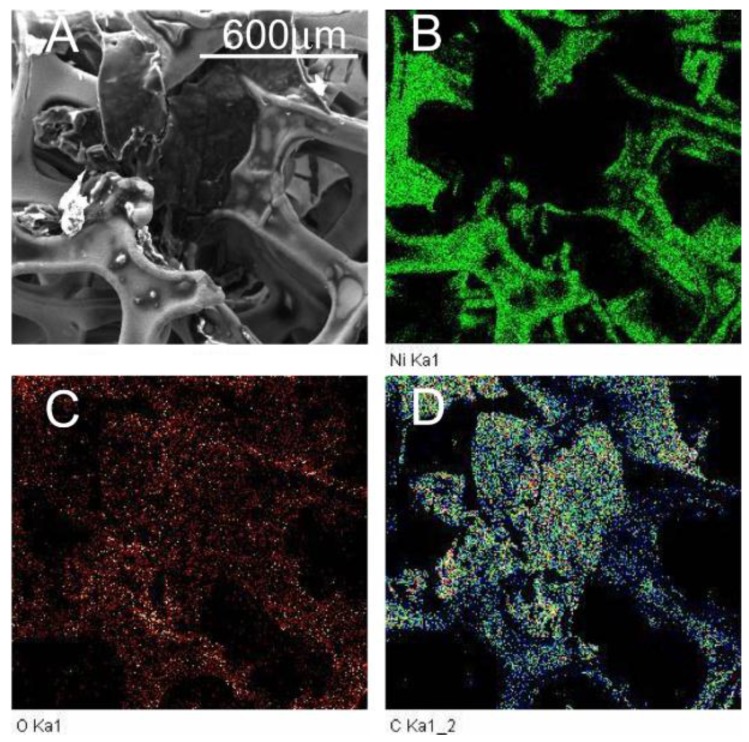
SEM image (**A**), chemical map of nickel (**B**), oxygen (**C**) and carbon (**D**) from a sample with 18 APA scans.

Figure 1A displays an ESEM chemical map of the sample subject to 12 APA scans. The image shows a characteristic APA processed RGON sample. The brightness of the elemental maps indicates particular element concentrations at each location. The carbon element map shows that there are areas on the map (Figure 1D) where this element has aggregated on the nickel foam. A visual comparison of Figure 1B,C shows that oxygen is concentrated in areas where nickel is present, rather than where the carbon is present. However, similar data from a sample processed with 18 plasma scans (Figure 2) exhibited a greater overlap between regions rich in both oxygen and carbon, suggesting that more oxygen was bound to carbon than in the sample with 12 scans of APA. This indicates that the reduction of GO to RGO is better in the sample with 12 scans than the one with 18 scans.

Table 1 summarizes the average carbon to oxygen ratios from the samples subjected to different numbers of plasma scans. The RGON surface after APA was not uniform and the variation in C:O ratios was high; however, the overall trend showed that the ratio of carbon to oxygen increased with the number of plasma scans. This experiment demonstrated that C:O ratios as high as 13.9:1 can be obtained using the APA technique. However, the chemical maps showed that the nature of oxygen bonds within samples may vary with the number of APA scans. For 10–16 scans, oxygen was prevalent in nickel-rich areas, whereas above that, it was more prevalent in carbon-rich areas. This suggests that there could be an optimum number of nitrogen plasma scans in the 10–18 scan range (180 °C to 280 °C temperature range) for optimized conversion of GO to RGO on the surface of the RGON sample.

**Figure 3:**
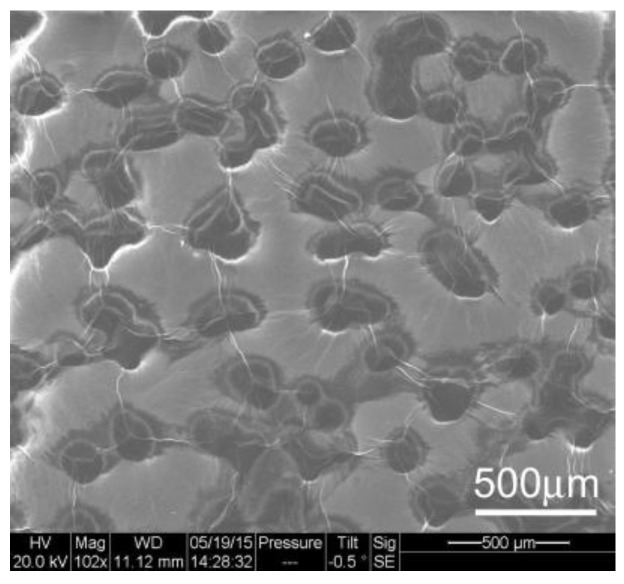
SEM image of unprocessed sample of nickel foam, soaked with graphene oxide and air dried.

**Figure 4:**
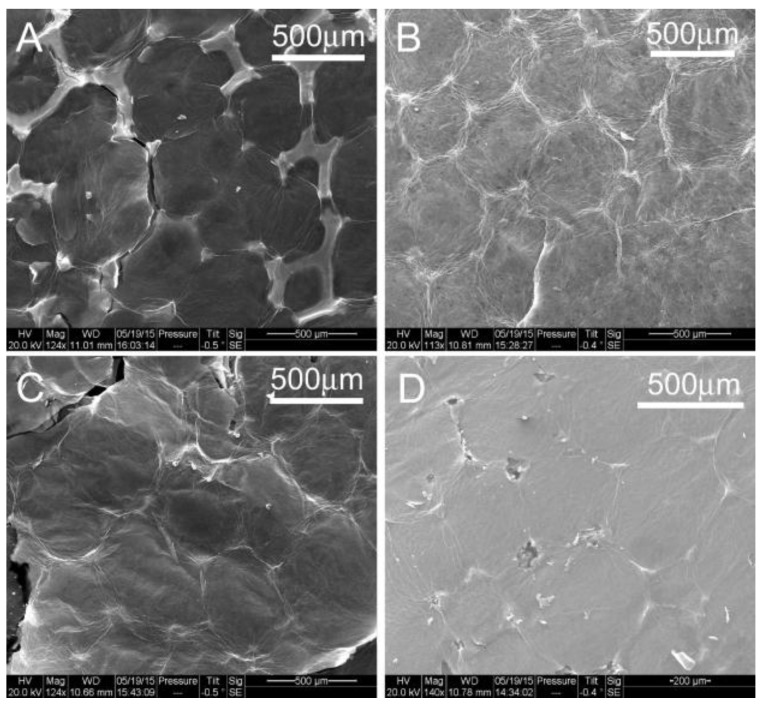
ESEM images of samples subject to FA at (**A**) 600 °C, (**B**) 700 °C, (**C**) 800 °C and (**D**) 900 °C, respectively.

Figure 3 displays the SEM image obtained from an unprocessed sample of nickel foam soaked with graphene oxide three times and air dried. The graphene oxide coats the surface of the nickel foam including the holes on the spongy structure. The film on the surface of the holes appears wrinkled and undulated. SEM images of the FA samples are displayed in Figure 4. The images indicate that the film spreads out and the surface is less wrinkled and stretched on the nickel foam with an increasing FA temperature.

**Figure 5:**
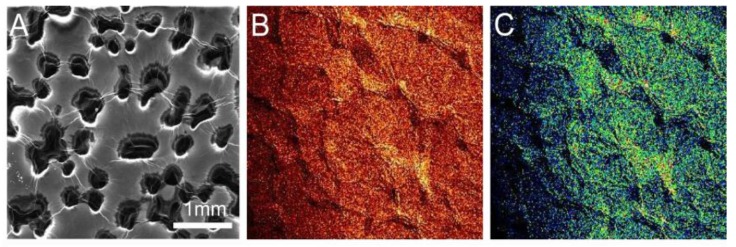
SEM image (**A**) and chemical map of carbon (**B**) and oxygen (**C**) distribution for an unannealed sample. Bright regions indicate regions where the element has a greater presence.

**Figure 6:**
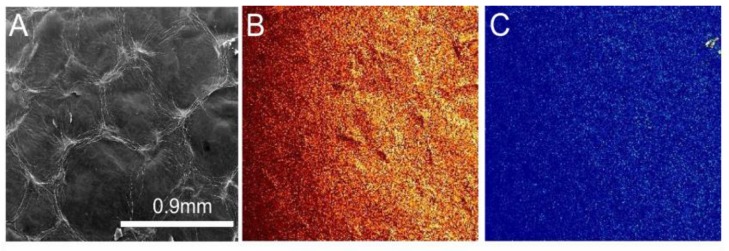
SEM image (**A**) and chemical map of carbon (**B**) and oxygen (**C**) distribution for an 800 °C annealed sample.

The bright regions at the edges of the RGO areas in the SEM images in Figure 4 indicate charge accumulation at the edges of the segments during ESEM data collection. The charge accumulated areas were absent on the unannealed samples, as seen in Figure 3. The accumulation of charge along boundary lines decreased with an increase in annealing temperatures. An image and the chemical map comparing carbon and oxygen distribution on the unannealed sample to one annealed at 800 °C are shown in Figure 5 and Figure 6, respectively. The reduction of GO is apparent in the distribution of carbon and reduced amount of oxygen in the annealed sample. The average pore lengths and widths obtained from SEM images of the nickel foam were 393.75 +/− 90.3 microns and 293.75 +/− 47.39 microns, respectively. During soaking, GO coated the region within and on the surface of the pores. After annealing, RGO was found to spread over the pores on the foam and form films on the surface of the foam. Figure 5A and Figure 6A contrast the appearance of the film before and after annealing, as it gets stretched over the holes on the surface of the nickel sponge, creating less wrinkled areas that are square millimeters in size.

The average ratio of carbon to oxygen, along with the standard deviation calculated from the atomic percentages, obtained from ESEM data are presented in Table 1. These numbers represent the average over at least two regions, varying in area from tens to hundreds of micrometers in size, for two separate, but identically processed, FA samples. It is apparent from the presented data that the C:O ratio increases with annealing temperature; however, the standard deviation also increases with annealing temperature, indicating increased non-uniformity for annealing above 800 °C.

The data obtained from ESEM data is collected to a depth of micrometers and over surface areas that are hundreds of micrometers or a millimeter squared. The variation in surface charge, observed in the SEM images of the RGON samples, processed by FA at various temperatures, raises the question as to whether the chemistry of the surface layers was different from the overall data that the ESEM provided. It was therefore interesting to look at the C:O ratio of the surface atomic layers, using XPS. Furthermore, XPS would confirm whether the type of carbon present was SP2 hybridized carbon, which is found in graphene. The purpose of obtaining the XPS data was to determine if the C:O ratio of the surface layers were similar to that within a few micrometers depth and if this correlated with capacitor performance.

### 2.2. X-ray Photoelectron Spectroscopy (XPS) Results

A Physical PHI Electronic-X Tool Model XPS was used with a resolution of 0.025 eV to analyze the binding energies of the surface layers of the processed samples, to determine if the carbon–oxygen (C–O) bonds were reduced and the SP2 hybridized carbon-carbon (C–C) bonds were prevalent. A binding energy of 284.8 eV is characteristic of C–C (SP2) bonds, whereas a binding energy of 285.5 eV indicates the presence of SP3 type carbon. Higher binding energies at 286 eV and about 288.5 eV are characteristic of C–O and C=O [19].

**Figure 7:**
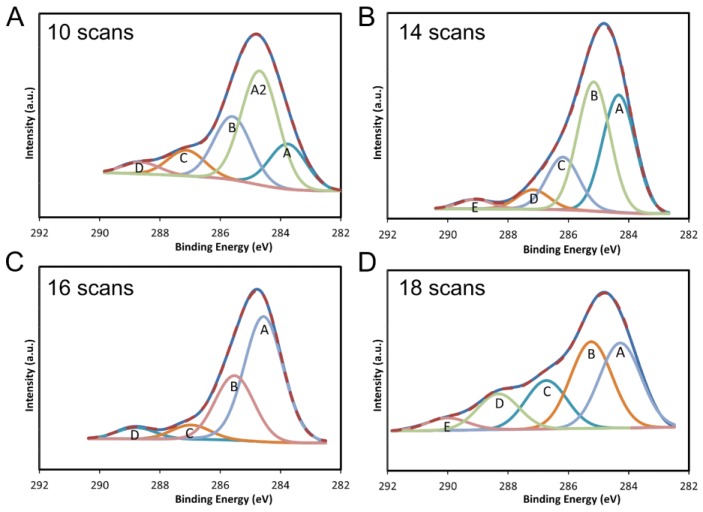
X-ray Photoelectron Spectroscopy (XPS) data from the unannealed sample and those subject to (**A**) 10, (**B**) 14, (**C**) 16 and (**D**) 18 scans of APA.

**Figure 8:**
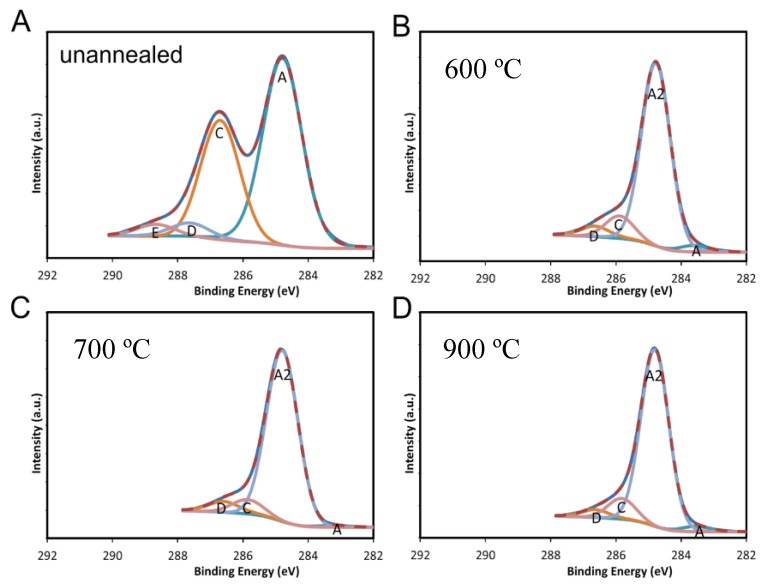
Comparison of the intensity of SP2 carbon peak intensity to C–O peak intensity from an (**A**) unannealed sample and after FA, at (**B**) 600 °C, (**C**) 700 °C and (**D**) 900 °C, for two hours.

The results from samples subject to 10, 14, 16, and 18 scans of APA are displayed in Figure 7. The XPS from the control sample without annealing is displayed in Figure 8A. The data from the control sample could be curve-fit to several peaks at binding energies that correspond to SP2 carbon (284.5 eV), SP3 carbon (285.2 eV), C–O bonds (286.3 eV) and C=O (288 eV) bonds. The best fits were obtained when a peak at 284.15 eV was included. The 0.3 eV shift in the SP2 carbon peak to the lower binding energy is attributed to the instrument. The ratio of the area of the SP2 carbon peak to the C–O peak was less than 2:1 and the ratio of SP2 to SP3 carbon was about 1:1.2 for the unannealed sample.

The XPS results from all the APA samples are summarized in Table 2. A quantity of ten scans of APA was found to dramatically reduce the C–O bonds in the surface layers, which are prominent in the unannealed sample (Figure 8A), and the binding energy at 284.8 eV, characteristic of C–C bond formation, was enhanced in intensity. The SP2 spectral signature intensity grew relative to the SP3 carbon signature when the number of APA scans increased from 12 to 16. This was accompanied by a reduction in the C–O signature. The XPS data from the sample that underwent 18 scans showed the SP2 carbon signature to be diminished considerably and the SP3 carbon signature, which has a slightly higher binding energy, was enhanced. The XPS data showed that an intermediate number of plasma scans is optimal for the reduction of oxygen and transformation of GO to RGO in the surface layers, whereas the ESEM results show that the C:O ratio increases with the number of scans. Thus, an intermediate number of scans should be used to optimize conductivity of the surface layers for the electrode.

The XPS spectra from the FA samples is presented in Figure 8B–D. The data displays strong SP2 carbon features, showing very good reduction of oxygen. Table 3 summarizes the data from all the FA samples.

Data from the FA sample processed at 600 °C shows the ratio of the area under the SP2 carbon peak to SP3 peak growing to above 14:1 and the ratio of the SP2 carbon peak to C–O peak becoming larger than 9:1. These ratios are increased relative to the unannealed sample, in which the ratio of SP2 to SP3 carbon was about 1:1.2, and the ratio of the area of the SP2 carbon peak to the C–O peak was less than 2:1. This indicates a removal of oxygen in the surface atomic layers of the RGON electrode by this process. In addition, the SP2 carbon peak narrows to about 0.6 eV in width after annealing, compared to 1 eV in the unannealed sample. The lower binding energy signature at 284.15 eV indicates the presence of defects, such as SP2 C–H or SP3 C–CH3 in the surface layers [23]. The sample annealed at 700 °C displayed a relative reduction in the SP2 carbon signature and an increase in the lower energy peak at 284.15 eV. The sample annealed at 900 °C, however, showed a higher intensity of carbon–oxygen bonds. This suggests that when higher temperatures are used for annealing, the surface layers allow oxygen to permeate more easily, and the conversion from GO to RGO is not facilitated in those layers of the RGON samples. This is in contrast to the ESEM data, which indicated that the material below the surface layers is reduced to RGO. This could affect the performance of a supercapacitor constructed with these samples as electrodes.

### 2.3. Fourier Transform Infrared Spectroscopy (FTIR) Results

**Figure 9:**
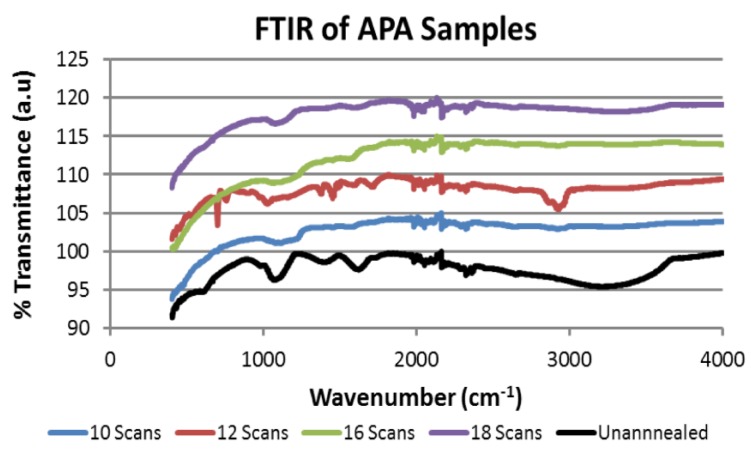
Fourier Transform Infrared Spectroscopy (FTIR) spectra for plasma annealed samples.

The ATR-FTIR analysis was performed to investigate the removal of water from the surface of the samples subject to APA. A Nicolet 50 infrared spectrometer (Thermo Fisher Scientific, Waltham, MA, USA) was used in reflection to collect data from the sample surface. Figure 9 summarizes the results obtained from samples that were annealed for 0 (unannealed), 10, 16, and 18 scans of APA in nitrogen gas. Our results were compared to those reported [24]. The characteristic O–H absorption signature at 3340 cm^−1^ was observed, along with the C–O absorption at 1067 cm^−1^, in the unannealed sample.

**Figure 10:**
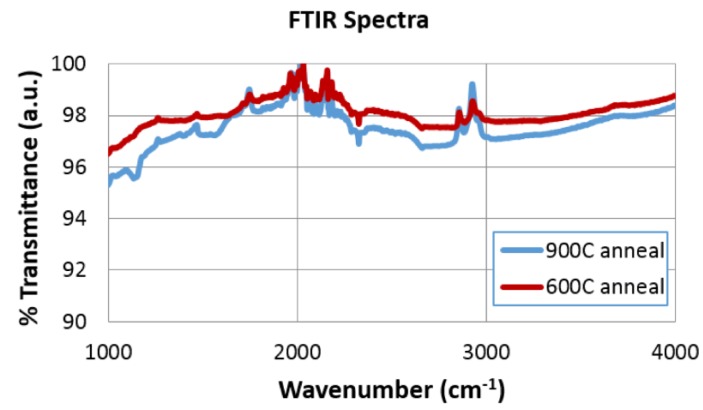
FTIR Spectra for thermal annealed samples.

The absorption at these frequencies was substantially reduced with an increasing number of scans. This is consistent with reports that RGO has no significant absorption in the frequency range above 3000 cm^−1^ [24]. However, the sample subjected to 18 scans showed a recovery of the OH absorption greater than the one subjected to 10 scans. This further supports the evidence provided by ESEM and XPS experiments that there is an intermediate number of scans that results in optimal reduction of GO on nickel foam electrodes. The ATR-FTIR results from the samples that underwent thermal processing at 600 °C and 900 °C (Figure 10) also showed a reduction in the OH absorption signature at 3400 cm^−1^ and the C–O signature at 1067 cm^−1^.

### 2.4. Raman Spectroscopy Results from APA Samples

**Figure 11:**
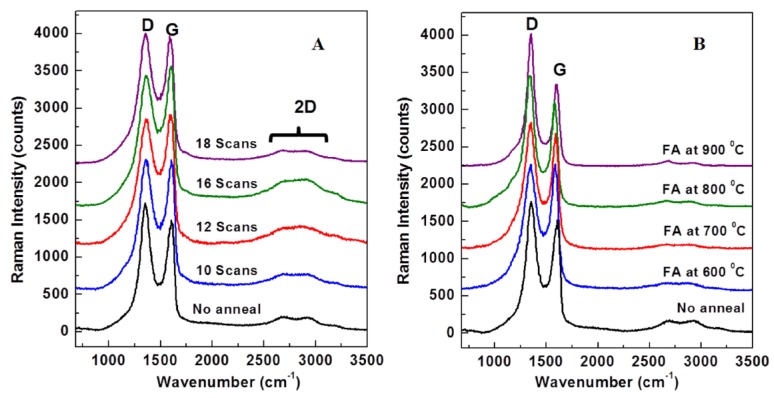
Raman spectra for (**A**) APA samples and (**B**) FA samples.

Raman spectroscopy was also employed to determine the extent of graphene oxide reduction when APA and FA were used. The Raman measurements were performed in a backscattering geometry with a confocal alpha 300R WITec system (WiTec, 300R, Ulm, Germany) equipped with a 532 nm Nd:YAG laser and a back-illuminated Charge Coupled Device (CCD) camera. A very low laser power of a few mW was used. Characteristic phonon modes, such as the D and G bands, were investigated, along with the signature of the second order process, involving two TO phonons near K, which is labeled 2D. The results are presented in Figure 11A,B for the samples processed with APA and FA, respectively. Appropriate background subtraction and normalization to the intensity of the D band were performed for all spectra. Also, the spectra were presented in the region of interest, namely between 680 cm^−1^ and 3500 cm^−1^, and vertically translated for easier visualization.

The observed D and G peaks at 1353 cm^−1^ and 1584 cm^−1^, respectively, as well as the 2D band at ~2700 cm^−1^ are in good agreement with their reported literature values [23,25]. In Figure 11A, the comparison of the sample that was not annealed, to one that had 10 scans of APA performed, shows an enhancement of the G peak. When the number of plasma scans was increased to 16, the G peak intensity again increased, showing a trend for oxygen removal. However, at 18 scans, the trend was reversed. This observation again supports the information obtained from the other techniques used to characterize these samples that an intermediate number of scans is optimal in reducing the surface GO on the samples. The Raman results for the samples undergoing FA at different temperatures—which are presented in Figure 11B—revealed an increase in the ratio of the intensity of the D peak to that of the G peak (ID/IG) indicating a recovery of SP2 domains in the material and a reduction of the surface GO for FA, up to 700 °C. The relative intensity of the G peak decreased for FA processing above that temperature, indicating that oxygen is more easily re-absorbed into the surface atomic layers at higher temperature of processing. This correlated with the XPS data, which showed higher oxygen contents for the 800 °C and 900 °C FA samples. X-ray diffraction also showed an optimal graphene signature below 800 °C. The slight increase in the intensity ratio of the 2D and G peaks (I2D/IG) with annealing temperatures, is attributed to the restoration of C–C interatomic distances and angles characteristic of graphene, as well as to smoothing of the films, which is observed in the ESEM images [24].The oxide functional groups in GO are bonded to the basal planes and, therefore, a reduction in oxygen will reduce the curvature of these planes and enhance the graphene-like Raman signature from the samples.

### 2.5. X-ray Diffraction (XRD) Results

A Rigaku 2000 system (Tokyo, Japan), with the monochromator tuned to Cu-Kα radiation, and a scintillation detector was used to collect X-ray diffraction data, with an accuracy of 0.02 degrees for the angle of diffraction. The interlayer spacing of graphite was 3.35 Å however, GO displayed an increase in the basal plane spacing that is due to functionalization of graphite with oxygen-containing groups [26]. In addition, d spacing of GO varied with water content in the sample and was considerably higher than that of graphite. X-ray diffraction data was collected from the unannealed sample and those subject to FA. The APA samples were non-uniform and X-ray diffraction data could not be obtained from them.

**Figure 12:**
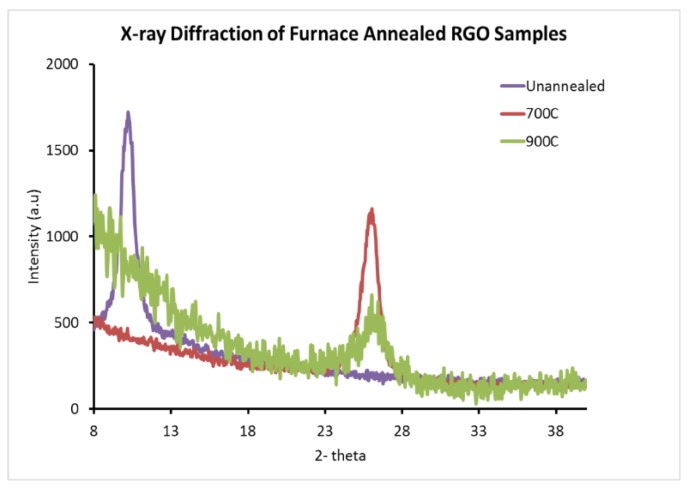
XRD data for furnace annealed samples.

X-ray data from the unannealed GO sample and that subject to FA at 700 °C and 900 °C is presented in Figure 12. The spectral signature at a 2-theta value of 10.04 degrees for the unannealed sample correlated to GO, with a humidity of 22.5%, with a corresponding d-spacing of 4.428 Å [25]. When furnace annealed, the GO structure disappeared and a broad peak at a 2-theta of around 26.12 degrees, characteristic of RGO, was seen. The spectral signature of graphite was not observed in the samples that were thermally processed. The RGO peak was found to have minimal full width at half maximum for the sample annealed at 700 °C. Our results are consistent with the results in reference [26], which show that above 600 °C, the spectral signature is shifted to higher angles, with a broadening of the peak. Our X-ray data shifted from 25.88 degrees for the 600 °C annealed sample, to 26.36 degrees for the 900 °C annealed sample. The full width at half maximum was slightly lower than that reported in reference 21 at 1.1 degrees for the 700 °C and the experimental d spacing decreased from 3.44 Å to 3.38 Å for the 600 °C to 900 °C range. The X-ray diffraction data was obtained from a large number of atomic layers and correlates with the ESEM data, which showed that oxygen is considerably reduced when data is averaged over hundreds of atomic layers.

### 2.6. Capacitance Measurements

The purpose of fabricating RGON electrodes was to construct electrical double layer capacitors (EDLC) and correlate the data obtained for the electrode material with performance of the capacitor [22]. The current APA samples could not be used as electrodes to construct capacitors, due to surface non-uniformity and mechanical fragility of the samples. Additional experiments are planned to improve the composite electrodes and measure the capacitance.

**Figure 13:**
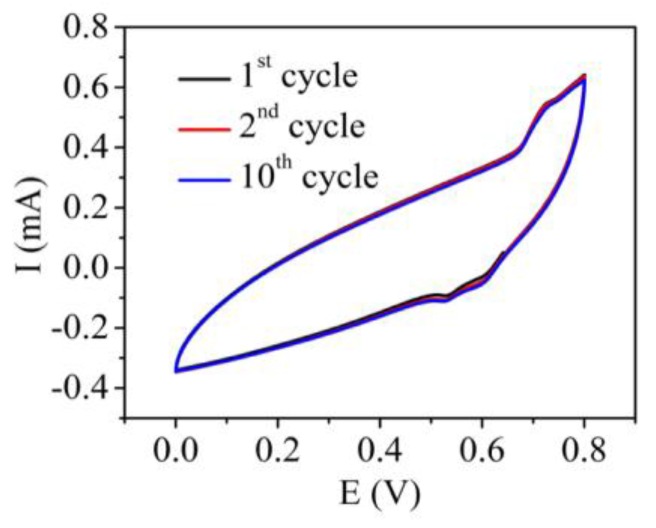
I–V data for ten cycles.

FA samples that were identically annealed at a specific temperature were used as electrodes to construct EDLCs, with Cellgard as a separator and potassium hydroxide as the electrolyte. The masses of the electrodes were carefully measured before soaking and after the thermal processing was completed. I–V data cycles were obtained from the capacitors constructed with samples annealed at various temperatures and they showed consistency in cycling. Figure 13 shows data for 10 cycles.

**Figure 14:**
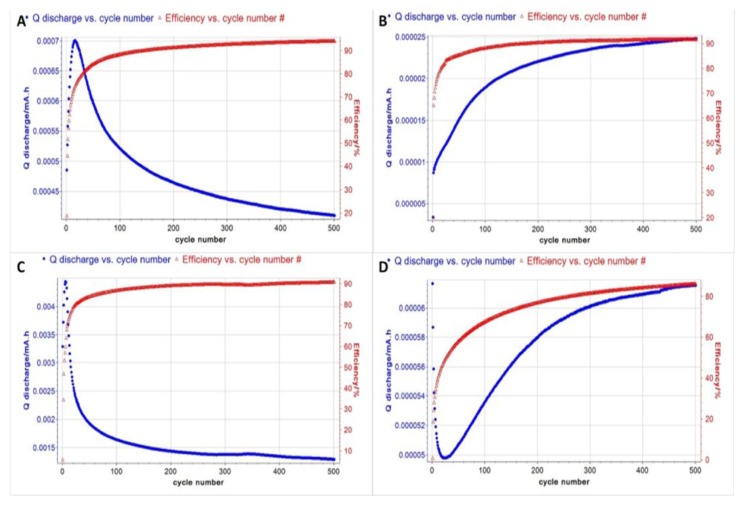
Constant current charging, discharging test for RGO-based electrodes with different annealing conditions: (**A**) 600 °C; (**B**) 700 °C; (**C**) 800 °C and (**D**) 900 °C. (The red line indicates efficiency vs. cycle number and the blue line discharge vs. cycle number).

Constant current charging and discharging tests were performed for the unannealed sample and those subjected to FA, at 600 °C, 700 °C, 800 °C and 900 °C. The data are displayed in Figure 14. The specific capacitance/unit mass was calculated using Equation (1).

(1) $$\frac{Capacitance}{Mass}=\frac{\left[Discharge\;Capacitance\;\left(Amp\cdot hours\right)\right]}{\left[Average\;Mass\times Voltage\right]}$$

The first results of the specific capacitance measurements are displayed in Table 4. As expected, the unannealed sample showed very low specific capacitance, most likely due to the low electrode conductivity, due to the GO. The best result of 33 F/g was obtained from the sample annealed at 600 °C. Electrodes employing CNTs have resulted in specific capacitances of 50 F/m^2^ [27]. Using SWCNT (Single Walled Carbon Nanotubes) networks, an EDLC capacitance of 180 F/g has been reported for power densities at 20 kW/kg, at energy densities in the 6.5–7 Wh/kg range, at 0.9 V, with a 7.5 M KOH electrolyte [28]. MnO_2_ and RuO_2_ nanoparticles deposited on carbon substrates have yielded specific capacitances as high as 1300 F/g [29,30]. The advantage of the method presented in this paper is the simplicity in sample preparation and efforts are underway to improve its performance.

These results correlate with data obtained by the various characterization methods. The ESEM images showed charging areas on the surface of the electrodes when annealed at 600 °C. These areas narrowed to thin lines at the boundaries, as the GO lost some of the wrinkles and spread on the surface of the nickel foam. The samples annealed at 800 °C and 900 °C showed very little charging. The XPS data showed that oxygen is removed on annealing at 600 °C and 700 °C. However, the surface atomic layers incorporated oxygen when samples were annealed at higher temperatures. Thus, although the ESEM C:O ratio was found to increase as annealing temperature increased, the electrode performance was not correlated with temperature. Rather, it is the surface layers that determine the performance of the electrodes in the capacitor. The supercapacitor made with 600 °C annealed electrodes had the lowest C:O ratio in the surface atomic layers, as indicated by the XPS data.

## 3. Materials and Methods

### 3.1. Atmospheric Plasma Annealing

**Figure 15:**
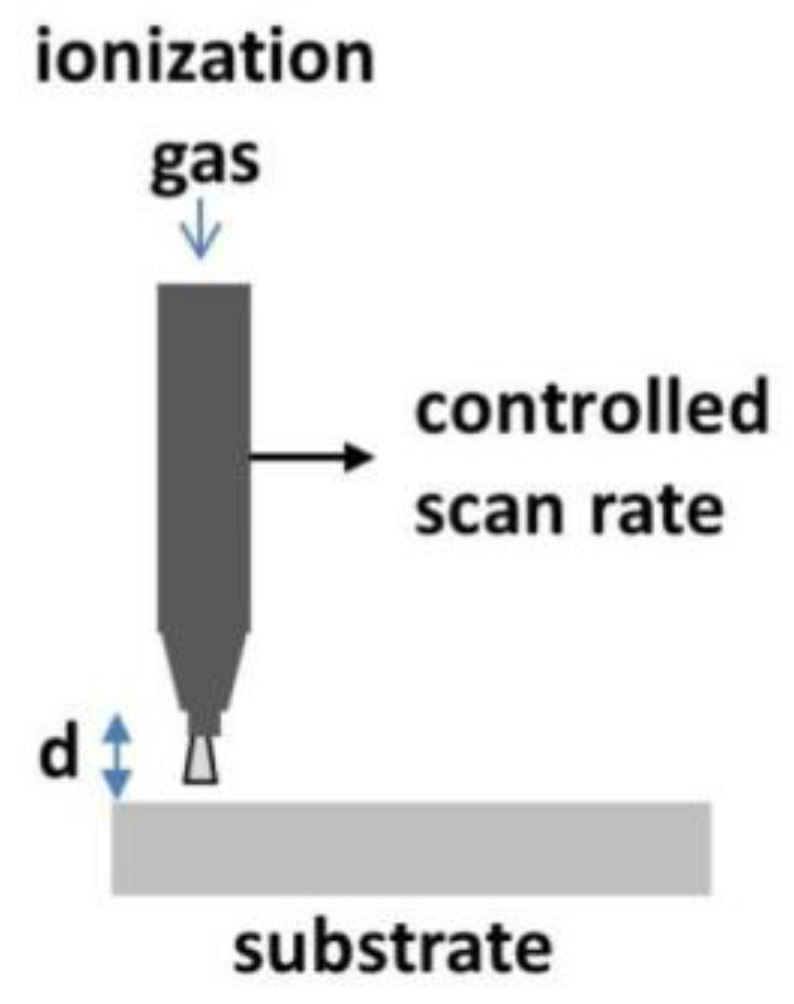
Plasma treatment process.

GO (4 mg/mL concentration) from Sigma–Aldrich and nickel foam sheets with a thickness of 1.7 mm from Novamet were used to fabricate the RGON electrodes. The foam sheets had roughly oval pores with an average length of 393.75 ± 90.03 microns and width of 293.75 ± 47.39 microns. Disks with 1.6 cm diameters were punched out, soaked in GO solution, and allowed to dry at room temperature. Figure 15 shows the scanning, DC-pulsed APA system, supplied by PlasmaTreat. Ionized nitrogen gas plasma was scanned over the fixed sample and the temperature of the backside of the sample was monitored. The number of plasma scans varied from 10 to 18. The diameter of the plasma jet was 0.5 cm and it was scanned at 10 m/min, with a gas flow rate of 1800 L/h with the power set to 1200 W. The temperature of the back of the sample opposite the incoming plasma jet was found to range from 180 °C to 280 °C when the plasma nozzle was maintained at a distance of 1 cm from the sample surface.

### 3.2. Furnace Annealing

RGON electrode samples were prepared for the purpose of furnace annealing in a manner similar to that done for APA. In the case of APA, the plasma treatment was done from the top surface and therefore the foam was only soaked once. Since FA processes the entire sample, the loading of the GO on nickel foam could be increased. The samples were soaked and air-dried three times, to increase the amount of GO present in the foam. The average mass of GO per disk of nickel foam was approximately 0.09 g after air drying. The samples were then subjected to thermal annealing at 600 °C, 700 °C, 800 °C or 900 °C for two hours in an atmosphere of 5% hydrogen and 95% argon. The ramp up rate was 5 °C/min. After annealing, the furnace was shut off and the samples were allowed to cool slowly.

### 3.3. Sample Characterization

ESEM data was obtained using a Quanta 200 s Environmental Scanning Electron Microscope system (Thermo Fisher Scientific, Waltham, MA, USA). ESEM data is collected over a depth of micrometers and is suitable to determine overall composition, chemical maps that show distribution of the elements and for image analysis. Images were obtained from an unannealed sample as well as the samples that were subject to APA or furnace annealing. Locations where charge would collect were also analyzed from the images. The ratio of carbon to oxygen was calculated by averaging values over 3 or more such areas for the samples. Elemental compositions, detailing the distribution of carbon, oxygen and nickel over a specified area, were determined at different locations on the sample. From this it was possible to identify where the oxygen was present in a given area. An overlay of a bright oxygen map in areas where the carbon map is bright would indicate the presence of both elements in that region. A reduction in the brightness of oxygen map in the areas where carbon is present would show a decrease in oxygen content where carbon is present. The non-uniformity of the APA samples permitted only trends to be observed and statistical averages and standard deviations were obtained for the FA samples.

In order to characterize the surface of the RGON films, X-ray photoelectron spectroscopy (Ulvac-Phi Inc., Chigasaki, Japan) was conducted, using a PHI VersaProbe I, with an ellipsoidal monochromater, LaB6 scanning electron source, and aluminum anode. Survey scans (binding energy range: 0–1350 eV, eV/step: 1, pass energy: 280 eV) and detailed spectra (eV/step: 0.1, pass energy: 55 eV) were measured using the XPS system. XPS data were analyzed using the MultiPak software package (v.9., Physical Electronics, Ulvac-Phi. Inc., Chigasaki, Japan). The experimental spectra were fitted using Gaussian–Lorentzian peaks (85:15 ratio), with each having the same full width at half maximum, as detailed by previous fitting models in literature [31]. A Rigaku Miniflex 600 system (Tokyo, Japan) with CuKα line was used to collect XRD data in the range 5°–35°, in steps of 0.04°. The data identified the diffraction pattern from GO and the method facilitated verification of a graphene-type signature after processing the samples using FA. A Nicolet 50 infrared spectrometer was employed to obtain ATR-FTIR data in the 500–4000 cm^−1^ frequency range. The purpose was to correlate -OH and C–O bond variation with processing parameters. A reduction in the intensity of these bond vibrations would indicate removal of water and oxygen. This was used as supplementary information to substantiate results from other techniques.

## 4. Conclusions

Subjecting RGON samples to APA using nitrogen ions resulted in the removal of oxygen from the composite. An analysis of the data from ESEM, XPS, Raman and FTIR demonstrated that that there may be an intermediate number of scans, between 10 and 18, that yields optimal reduction. Although the substrate temperature of nickel foam was low, at 180–280 °C, it is possible that the surface of the sample attained a higher temperature. Researchers at Stanford University have reported that N-doping can occur at temperatures of about 300 °C [32]. In addition, they have reported a better reduction of oxygen when their samples were subsequently annealed with ammonia plasma. Chemical doping of melamine in GO has also assisted in the synthesis of RGO by thermal processing. The catalytic activity of nitrogen in assisting this process has been explored [33]. We believe the nitrogen ion plasma applied to graphene oxide in air could have caused temporary nitrogen doping. No nitrogen signature was observed in the XPS data collected after annealing, indicating that the assist in the transformation was simply catalytic. Our observations therefore conclude that temporary doping of nitrogen in GO could enhance the reduction process and assist the formation of RGO, which should enhance conductivity of the electrode. However, our experiments showed that the APA method results in incomplete reduction of GO, since the annealing process is performed at atmospheric pressure, with exposure to air during the treatment. Shrouding the plasma jet to reduce the oxygen content in the area surrounding the substrate may improve the degree of reduction and result in better RGO formation. Additionally, by purely exposing the substrates to the plasma for specific time intervals, as opposed to scanning, may increase exposure to the radicals produced by plasma and may improve the degree of reduction. The samples from the APA experiment had non-uniform surfaces and fabrication of capacitors was not possible. Further experiments are necessary to improve the quality and uniformity of the electrode samples.

RGON formed by FA processing at the 600–900 °C range in hydrogen–argon atmospheres demonstrated good reduction of GO to RGO. The surface of the film showed more uniformity and less distinct regions stretched across the nickel foam where the film was present. Charging occurred at the edges of the RGO areas during ESEM data collection, indicating lower conductivity across boundaries of the regions. Furthermore, higher temperatures of annealing resulted in surface oxygen incorporation. With introduction of oxygen, conductivity decreased as GO content increased. Supercapacitors can be constructed using these samples as electrodes. A specific capacitance of 33 F/g was obtained for the sample annealed at 600 °C. It provided an indication that the reintroduction of oxygen on the surface deteriorates capacitor performance. Additional experiments are planned to improve the adhesion of the RGO to the nickel foam and construct the capacitors with different electrolytes to improve the values of specific capacitance.

## Figures and Tables

**Table 1:** Carbon to Oxygen Ratio from ESEM data.

APA Samples		Furnace Annealed (FA) Samples—Trial 1,2,3		
# Scans	Average C:O	Annealing Temperature	Average C:O	Standard Deviation of C:O
10	0.9	Not Annealed	1.04	0.12
12	2.8	600 °C	14.11	2.81
14	1.4	700 °C	16.03	2.10
16	9	800 °C	22.17	5.77
18	13.9	900 °C	35.06	9.58

**Table 2:** XPS results for APA scanned samples.

Attribute	Binding Energy (eV)	%	Attribute	Binding Energy (eV)	%
**10 scans**			**12 scans**		
A: C–C	283.98	17.38	A: C–C	284.34	35.85
A2: C–C	284.87	43.82	B: C=C	285.35	28.86
B: C=C	285.74	24.4	C: C–O–C	286.67	17.61
C: C–O–C	287.15	9.88	D: C=O	287.95	10.56
D: C=O	288.64	4.52	E: O–C=O	289.12	7.13
**14 scans**			**16 scans**		
A: C–C	284.35	35.42	A: C–C	284.56	59.24
B: C=C	285.16	38.92	B: C=C	285.53	29.17
C: C–O–C	286.18	16.62	C: C–O–C	286.97	6.45
D: C=O	287.7	6.15	D: C=O	288.77	5.14
E: O–C=O	289.09	2.88	-	-	-
**18 scans**			**20 scans**		
A: C–C	284.28	31.61	A: C–C	284.62	60.76
B: C=C	285.24	32.22	B: C=C	285.22	32.79
C: C–O–C	286.73	18.01	C: C–O–C	286.64	6.45
D: C=O	288.33	13.32	-	-	-
E: O–C=O	290.01	4.84	-	-	-

**Table 3:** The overall percentage of C to O ratio in the FA samples from XPS data.

Temperature (°C)	Avg. % °C	Uncertainty %	Avg. % O	Uncertainty %	Avg C/O	Uncertainty %
Unannealed	69.6	1.2	29.3	0.8	2.4	0.1
600	93.2	0.2	6.3	0.3	14.8	0.7
700	90.4	1.9	8.8	1.7	10.3	2.3
800	85.6	1.0	13.1	1.0	6.5	0.6
900	84.9	0.9	13.1	0.8	6.5	0.5

**Table 4:** Specific capacitance for supercapacitors built with FA samples used as electrodes.

Temperature (°C)	Capacitance (F/g)
Unannealed	0.45
600	33
700	2.8
800	1.9
900	0.6

## References

[1] Conte M., Genovese A., Ortenzi F., Vellucci F. (2014). Hybrid battery-supercapacitor storage for an electric forklift: A life-cycle cost assessment. J. Appl. Electrochem..

[2] Hong S.Y., Kim Y., Park Y., Choi A., Choi N.-S., Lee K.T. (2013). Charge carriers in rechargeable batteries: Na ions vs. Li ions. Energy Environ. Sci..

[3] Zhang J., Jiang J., Li H., Zhao X.S. (2011). A high-performance asymmetric supercapacitor fabricated with graphene-based electrodes. Energy Environ. Sci..

[4] Zhang S., Xiong R., Zhou X. (2015). Comparison of the topologies for a hybrid energy-storage system of electric vehicles via a novel optimization method. Sci. China Technol. Sci..

[5] Simon P., Gogotsi Y. (2008). Materials for electrochemical capacitors. Nat. Mater..

[6] López-Naranjo E.J., González-Ortiz L.J., Apátiga L.M., Rivera-Muñoz E.M., Manzano-Ramírez A. (2016). Transparent Electrodes: A Review of the Use of Carbon-Based Nanomaterials. J. Nanomater..

[7] Tung V.C., Chen L.-M., Allen M.J., Wassei J.K., Nelson K., Kaner R.B., Yang Y. (2009). Low-temperature solution processing of graphene-carbon nanotube hybrid materials for high-performance transparent conductors. Nano Lett..

[8] Chen T., Dai L. (2013). Carbon nanomaterials for high-performance supercapacitors. Mater. Today.

[9] Signorelli R., Ku D.C., Kassakian J.G., Schindall J.E. (2009). Electrochemical double-layer capacitors using carbon nanotube electrode structures. Proc. IEEE.

[10] Pan H., Li J., Feng Y.P. (2010). Carbon nanotubes for supercapacitor. Nanoscale Res. Lett..

[11] Yi M., Shen Z. (2015). A review on mechanical exfoliation for the scalable production of graphene. J. Mater. Chem. A.

[12] Zhao L., Rim K.T., Zhou H., He R., Heinz T.F., Pinczuk A., Flynn G.W., Pasupathy A.N. (2011). Influence of copper crystal surface on the CVD growth of large area monolayer graphene. Solid State Commun..

[13] Mohan V.B., Brown R., Jayaraman K., Bhattacharyya D. (2015). Characterisation of reduced graphene oxide: Effects of reduction variables on electrical conductivity. Mater. Sci. Eng. B Solid-State Mater. Adv. Technol..

[14] Chen Y., Fu K., Zhu S., Luo W., Wang Y., Li Y., Hitz E., Yao Y., Dai J., Wan J. (2016). Reduced graphene oxide films with ultrahigh conductivity as Li-ion battery current collectors. Nano Lett..

[15] Gao J. (2016). Free-standing reduced graphene oxide paper with high electrical conductivity. J. Electron. Mater..

[16] Jin S., Gao Q., Zeng X., Zhang R., Liu K., Shao X., Jin M. (2015). Effects of reduction methods on the structure and thermal conductivity of free-standing reduced graphene oxide films. Diam. Relat. Mater..

[17] Stoller M.D., Park S., Yanwu Z., An J., Ruoff R.S. (2008). Graphene-Based ultracapacitors. Nano Lett..

[18] El-Kady M.F., Strong V., Dubin S., Kaner R.B. (2012). Laser Scribing of High-Performance and Flexible Graphene-Based Electrochemical Capacitors. Science.

[19] Lee S.W., Mattevi C., Chhowalla M., Sankaran R.M. (2012). Plasma-assisted reduction of graphene oxide at low temperature and atmospheric pressure for flexible conductor applications. J. Phys. Chem. Lett..

[20] Zheng C., Zhang J., Zhang Q., You B., Chen G. (2015). Three dimensional Ni foam-supported graphene oxide for binder-free pseudocapacitor. Electrochim. Acta.

[21] Luan V.H., Tien H.N., Hoa L.T., Hien N.T.M., Oh E.-S., Chung J., Kim E.J., Choi W.M., Kong B.-S., Hur S.H. (2013). Synthesis of a highly conductive and large surface area graphene oxide hydrogel and its use in a supercapacitor. J. Mater. Chem. A.

[22] Cao X., Shi Y., Shi W., Lu G., Huang X., Yan Q., Zhang Q., Zhang H. (2011). Preparation of novel 3D graphene networks for supercapacitor applications. Small.

[23] Yang D., Velamakanni A., Bozoklu G., Park S., Stoller M., Piner R.D., Stankovich S., Jung I., Field D.A., Ventrice C.A. (2009). Chemical analysis of graphene oxide films after heat and chemical treatments by X-ray photoelectron and Micro-Raman spectroscopy. Carbon N. Y..

[24] Yamada Y., Yasuda H., Murota K., Nakamura M., Sodesawa T., Sato S. (2013). Analysis of heat-treated graphite oxide by X-ray photoelectron spectroscopy. J. Mater. Sci..

[25] Cuong T.V., Pham V.H., Tran Q.T., Hahn S.H., Chung J.S., Shin E.W., Kim E.J. (2010). Photoluminescence and Raman studies of graphene thin films prepared by reduction of graphene oxide. Mater. Lett..

[26] Blanton T., Majumdar D. (2013). Characterization of X-ray Irradiated graphene oxide coatings using X-ray diffraction, X-ray photoelectron spectroscopy, and atomic force microscopy. CPDS-Int. Centre Diffr. Data.

[27] Chen J.H., Li W.Z., Wang D.Z., Yang S.X., Wen J.G., Ren Z.F. (2002). Electrochemical characterization of carbon nanotubes as electrode in electrochemical double-layer capacitors. Carbon N. Y..

[28] An K.H., Kim W.S., Park Y.S., Moon J.M., Bae D.J., Lim S.C., Lee Y.S., Lee Y.H. (2001). Electrochemical properties of high-power supercapacitors using single-walled carbon nanotube electrodes. Adv. Funtional Mater..

[29] Sugimoto W., Iwata H., Yasunaga Y., Murakami Y., Takasu Y. (2003). Preparation of ruthenic acid nanosheets and utilization of its interlayer surface for electrochemical energy storage. Angew. Chemie Int. Ed..

[30] Zhao Y.-Q., Zhao D.-D., Tang P.-Y., Wang Y.-M., Xu C.-L., Li H.-L. (2012). MnO2/graphene/nickel foam composite as high performance supercapacitor electrode via a facile electrochemical deposition strategy. Mater. Lett..

[31] De Giglio E., Cometa S., Satriano C., Sabbatini L., Zambonin P.G. (2009). Electrosynthesis of hydrogel films on metal substrates for the development of coatings with tunable drug delivery performances. J. Biomed. Mater. Res. A.

[32] Du D., Li P., Ouyang J. (2015). Nitrogen-doped reduced graphene oxide prepared by simultaneous thermal reduction and nitrogen doping of graphene oxide in air and its application as an electrocatalyst. ACS Appl. Mater. Interfaces.

[33] Li X., Wang H., Robinson J.T., Sanchez H., Diankov G., Dai H. (2009). Simultaneous nitrogen doping and reduction of graphene oxide. J. Am. Chem. Soc..

